# Supplementary material for: Quantifying benefits of renewable investments for German residential Prosumers in times of volatile energy markets
Source: Nat Commun. 2024 Sep 18;15:8206. doi: 10.1038/s41467-024-51967-6 (PMC11410939; doi:10.1038/s41467-024-51967-6)
Supplement: Supplementary file 1 — Supplementary Information [file 41467_2024_51967_MOESM1_ESM.pdf]

1. Component modelling

The investment costs for photovoltaic (PV), home storage system (HSS), heat pump (HP), and hot water storage (HWS) are modelled in dependence of the scenario year. For PV systems and HSS investment costs have increased during the energy crisis [1]. Moreover, for these components we make the assumption that investment costs in 2023 are identical to those in 2022. For 2030, for solar PV installations an average cost reduction of 2% per year from 2022 on is considered, according to [2]. For HSS investment costs are projected to decline by at least 54% from 2016 towards 2030 [3]. Considering an average system price (exclusive inverter) of 1,364 €/kWh for an HSS in 2016 [4, 5], the price for 2030 is calculated to 627 €/kWh accordingly. For battery and PV inverters, we assume a constant price of 100 €/kW for all scenarios, derived from [6]. For 2020 air-source HP investment costs we assume a value of 1,856 €/kW$_{th}$, which is the average value for 2019 reported in [7] (with a conversion factor of 0.88 from USDollar to euros). In addition, it is assumed that there is a general price decrease for HP and a technology advance towards 2030, according to [8]. Furthermore, for HP investments we do not consider additional costs for exchange of radiators, window replacement, or additional insulation of the building. Recent studies show that for a substantial share of German buildings a HP can be installed without these additional measures [9, 10]. For all components, fixed annual costs for operation and maintenance are stated as percentages of the total initial investment costs.

The PV potential of German households depends on the regional topology and is higher on the countryside than in urban settlements [11]. Therefore, a smaller (8.7 kWp) and a larger (13.7 kWp) solar PV-systems are modelled to account for a typical range of households. For the HSS system, we do only consider one size (9.4 kWh). This is chosen as Figgener et al. [1] find that on average home owners purchase this size of HSS which is close to the size of the average PV system power. Furthermore, the purchase of an HSS is mainly driven by ideological reasons with regard to the energy transition and the striving for self-sufficiency rather than by economics [4]. For heating, most single-family homes (SFH) in Germany have a combined central system that supplies space heating (SH) as well as hot water (HW) for direct use through a boiler [12]. The typical size for a combined thermal storage in combination with a gas-fired boiler is assumed to be 25 kWh [13]. Therefore, for thermal storage we model a combined tank with a size of 25 kWh for SH and HW supply, both for a SFH with gas boiler (GB) and air-source HP. For modelling of air-source HP, a variable coefficient of performance (COP) is considered (average 3.99). It is calculated according to the formula in (1) with T$_{source}$ as the variable ambient air temperature [14]. For simplification reasons, we assume that an average supply temperature T$_{sink}$ of $50°C$ is sufficient for all household configurations [7]. The quality grade η_{HP} of the air-source HP is chosen to be 0.4, according to [14]. In addition, for HP installations the sizes are separately optimized for each household configuration. The electric top-up coil (TC) is only optimized for scenarios including a HP to cover high peaks in heat demand. For storage components, self-discharge losses of 0.17% per day for HSS [15] and 4.1% per day for thermal storage [16] are accounted for in the modelling. Another parameter to be set for storage is the energy-to-power (E2P) ratio. For HSS we choose a typical value of two hours [5] whereas for HWS it is set to 0.25 hours with the assumption that power availability is not critical for a thermal buffer storage.

$$COP = \frac{T_{sink} + 273.15°C}{T_{sink} - T_{source}} \cdot \eta_{HP} \quad T_{sink} = 50°C, \eta_{HP} = 40\% \tag{1}$$

In contrast to the operation of renewable components the manufacturing accounts for substantial greenhouse gas (GHG)-emissions as it requires substantial amounts of materials and use of energy. For solar PV an emission factor of 810 kg CO2eq./kWp is taken from [17] which accounts for modules manufactured in China with high shares of fossil electricity. Only for the future 2030 scenario we assume that a substantial solar PV manufacturing can be established in Europe and the emission factor decreases to 480 kg CO2eq./kWp [17]. For battery manufacturing, in a worst case assumption 106 kg CO2eq./kW are taken from [18]. For the heating components, Naumann et al. [19] perform a life cycle assessment (LCA) analysis between air source heat pumps and condensing GBs. For an air source heat pump the study estimates life cycle emissions of 24.4 gCO2eq./MJ of heat of which 3% can be assigned to manufacturing. With the total energy supplied over 20 years of operation and an output size of 5 kW$_{th}$ of the heat pump, also taken from [19], the emission factor is calculated to 163 kg CO2eq./kWp. For the GB no relevant emissions during manufacturing are taken into account [19]. The emission factor for inverters is calculated from the solar PV only case in Krebs et al. [20]. By dividing the manufacturing related emission factor of a component by its service life a yearly factor can be calculated.

2. Framework for Optimizing Sector-Coupled Urban Energy Systems (FOCUS) Framework

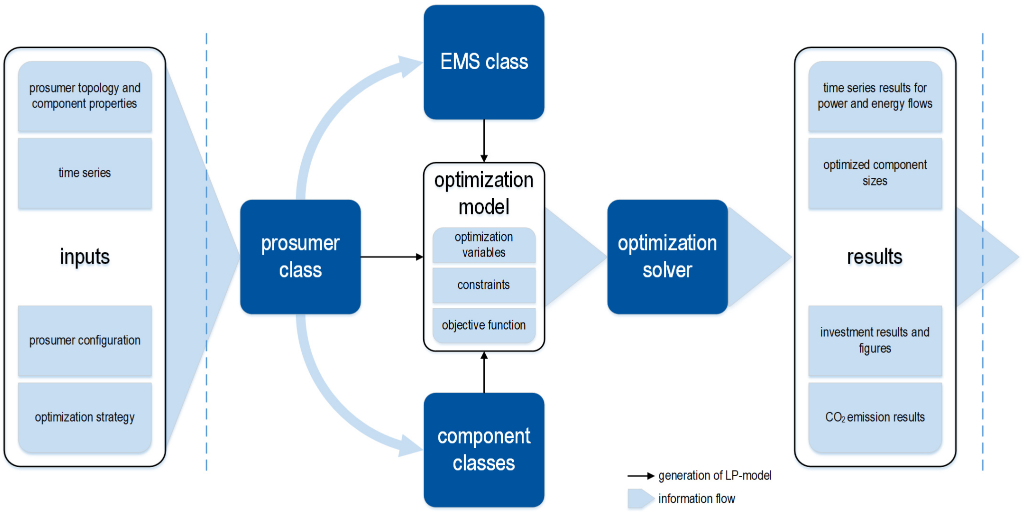

Figure 1: Visualization of the information flow within the Prosumer model of the FOCUS Framework [21] for energy system optimization developed at RWTH Aachen University.

3. Rolling horizon procedure

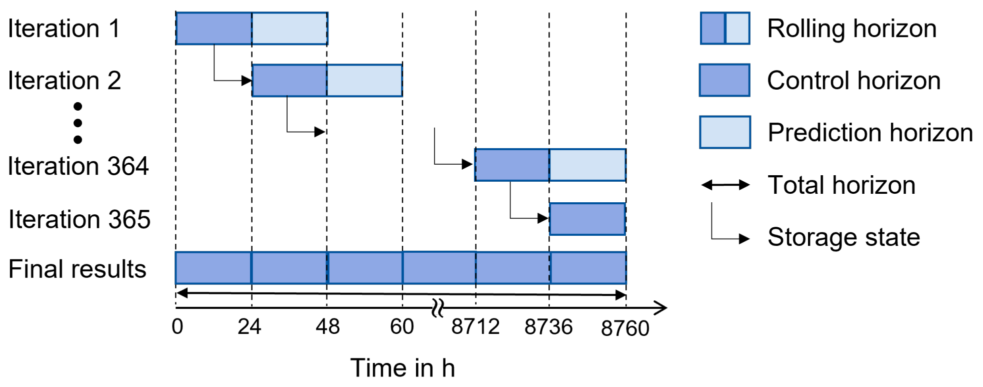

Figure 2: Visualization of the rolling horizon method with predictions that is used to find a lower threshold for the optimal solution.

Table 1: Prediction methods used for the rolling horizon approach.

Time series	prediction method	assumption
electricity demand	same hour last week	typically the electricity consumption is similar on the same weekdays
thermal demand	same hour last day	corresponds to air temperature which might change within a few days
electric vehicle (EV) demand	same hour last week	typically the driving schedule is similar on the same weekdays
solar irradiation	same hour last day	usually the weather can change within a few days
air temperature	same hour last day	usually the weather can change within a few days

4. Modelling data

Table 2: Used input profile data. The data is resampled to meet the required resolution of 15 min for the optimization.

Type	Origin	Resolution	Year	Description	Reference
electricity consumption	measured	1min	2010	real life measurements of 77 typical households at TU Berlin, profile 17	[22]
SH and HW consumption	generated	15min	generic	generated with synPro demand generator of Fraunhofer ISE with Energy Charts	[23]
electricity prices (Day Ahead Auction)	stock exchange	1h	2023	provided by Fraunhofer ISE	[24]
irradiance	generated	1h	2019	average values for Germany generated with renewables.ninja	[25, 26, 27]
temperature	measured	1h	generic	test reference years (TRY) by DWD	[28]
EV consumption	generated	1min	generic	internal simulation tool at ISEA RWTH Aachen	[29]
equivalent emissions electricity grid	generated	1h	2019	provided by Agorameter	[30]

Table 3: Overview of modeled components that are relevant for the household topologies that are illustrated in Figure **??**. All prices indicated are exclusive of value added tax (VAT) and subsidies.

Generating and conversion components										
Component	Year	Invest. costs		Fixed OPEX	Service life	Size	Efficiency (one way)		Emission factor (kg CO2-eq./kW)	References
PV system (excl. inverter)	2020	1005	€/kWp	1.0 %	32 a	8.7/13.7 kWp	variable			[2, 31, 32, 33, 1, 17]
	2021	948	€/kWp						China: 810	
	2022	1048	€/kWp						China: 810	
	2023	1048	€/kWp						China: 810	
	2030	856	€/kWp						EU: 480	
air source HP	2020	1856	€/kW$_{th}$	1.8 %	20 a	[0,30] kW$_{th}$	var. COP (avg: 3.9)		163	[7, 34, 32, 8, 35, 19]
	2021	1830	€/kW$_{th}$							
	2022	1811	€/kW$_{th}$							
	2023	1803	€/kW$_{th}$							
	2030	975	€/kW$_{th}$							
GB	all	175	€/kW$_{th}$	3.9 %	20 a	[0,30] kW$_{th}$	96 %		Not considered	[35, 36, 32, 37, 19]
electric TC	all	40	€/kW$_{th}$	2.0 %	20 a	[0,20] kW	98 %		Not considered	[14, 37]
inverter PV	all	150	€/kWp	1.0 %	15 a	8.7/13.7 kW	97 %		75	[6, 38, 39, 40, 41, 20, 42]
inverter battery	all	100	€/kW	1.0 %	15 a	4.7 kW	95 %		75	[6, 38, 39, 40, 43, 44, 20, 42]
**Storage components**										
Component	Year	Invest. Costs		Fixed OPEX	Service life	Size	Round-trip efficiency	E2P ratio	Emission factor (kg CO2-eq./kWh)	References
HSS (excl. inverter)	2020	980	€/kWh	1.0 %	15 a	9.4 kWh	96 %	2 h	106	[44, 1, 18, 42]
	2021	870	€/kWh							
	2022	1147	€/kWh							
	2023	1147	€/kWh							
	2030	627	€/kWh							
thermal storage combined	2020	35	€/kWh$_{th}$	0.0 %	30 a	25 kWh	98 %	0.25 h	Not considered	[8, 13, 37]
	2021	34	€/kWh$_{th}$							
	2022	33	€/kWh$_{th}$							
	2023	32	€/kWh$_{th}$							
	2030	23	€/kWh$_{th}$							

5. Evaluation of additional key performance indicator (KPI)s

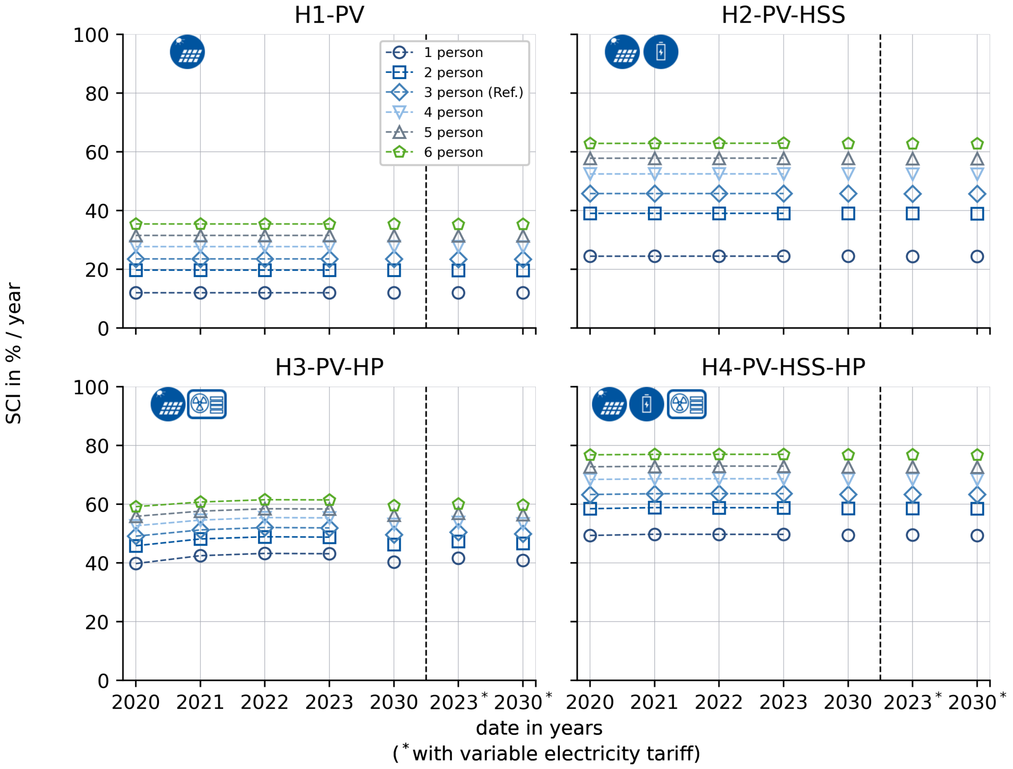

(a) Variation of number of residents, for a building constructed between 1979 and 1990.

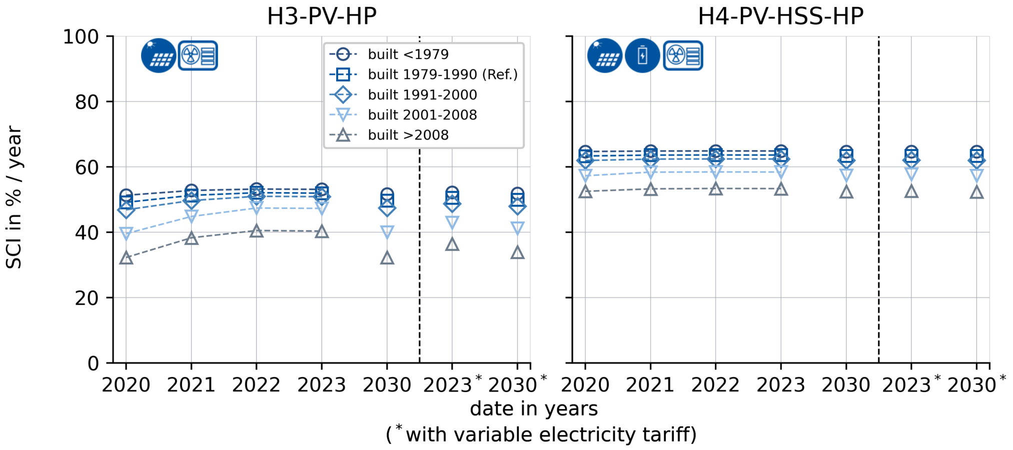

(b) Variation of years of construction of a building, for a three-person household.

Figure 3: self-consumption index (SCI) that is reached for different renewable SFH topologies. For selected *years**, variable instead of fixed electricity tariffs are applied.

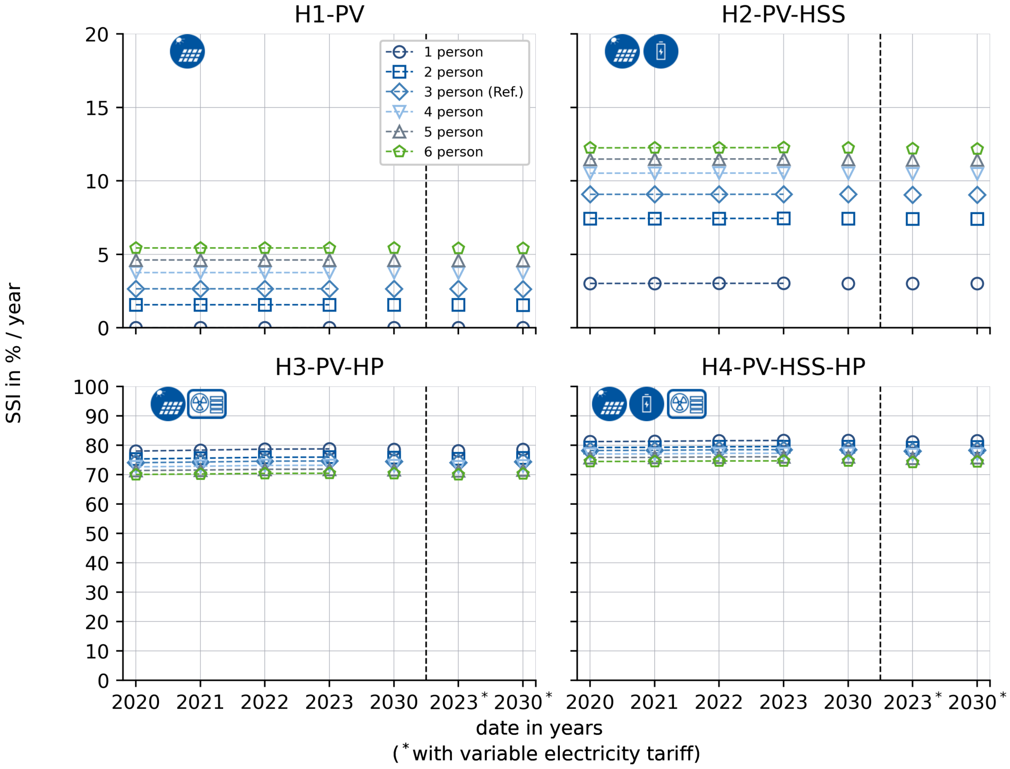

(a) Variation of number of residents, for a building constructed between 1979 and 1990.

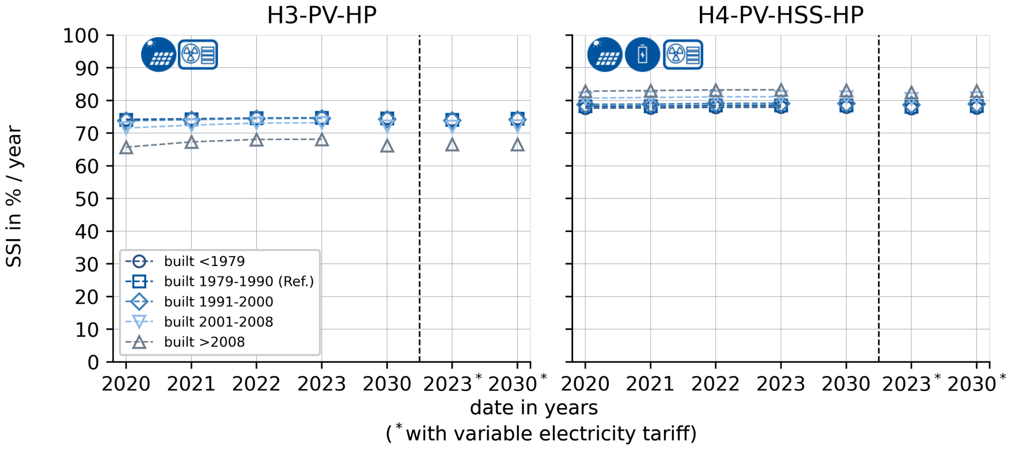

(b) Variation of years of construction of a building, for a three-person household.

Figure 4: self-sufficiency index (SSI) that is reached for different renewable SFH topologies. For selected $years^*$, variable instead of fixed electricity tariffs are applied.

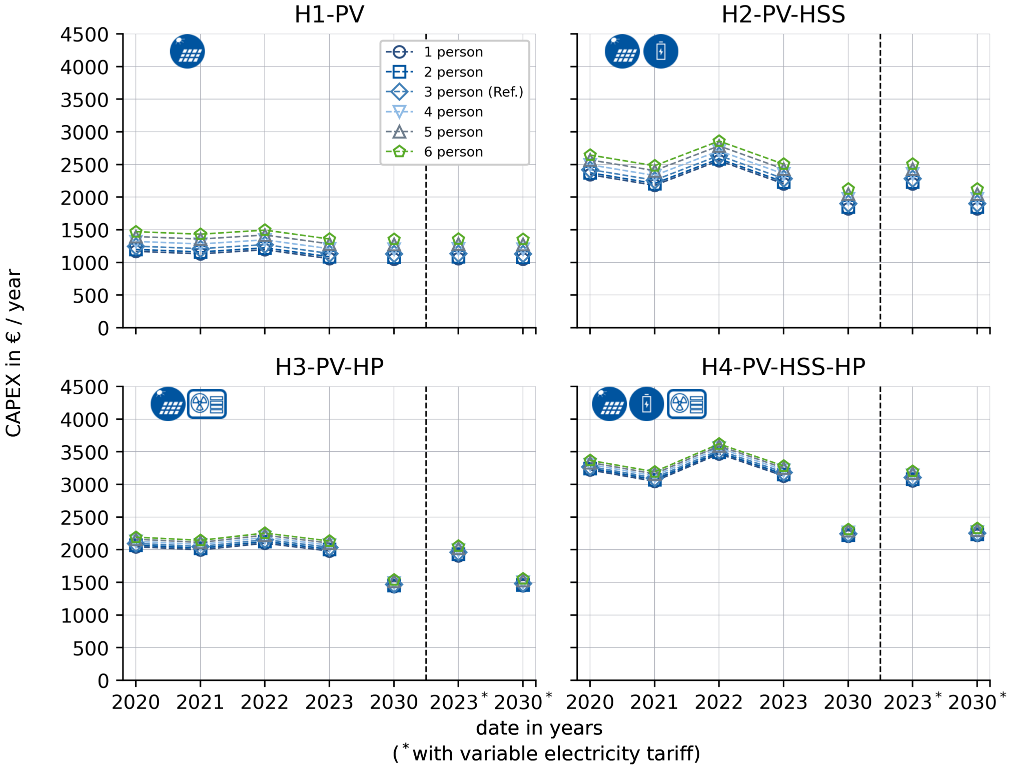

(a) Variation of number of residents, for a building constructed between 1979 and 1990.

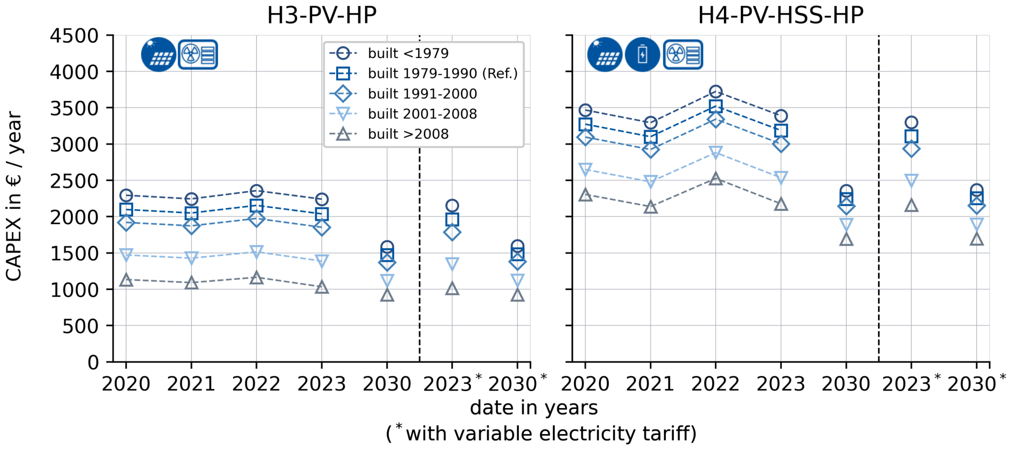

(b) Variation of years of construction of a building, for a three-person household.

Figure 5: capital expenditure (CAPEX) for different renewable SFH topologies. For selected *years**, variable instead of fixed electricity tariffs are applied.

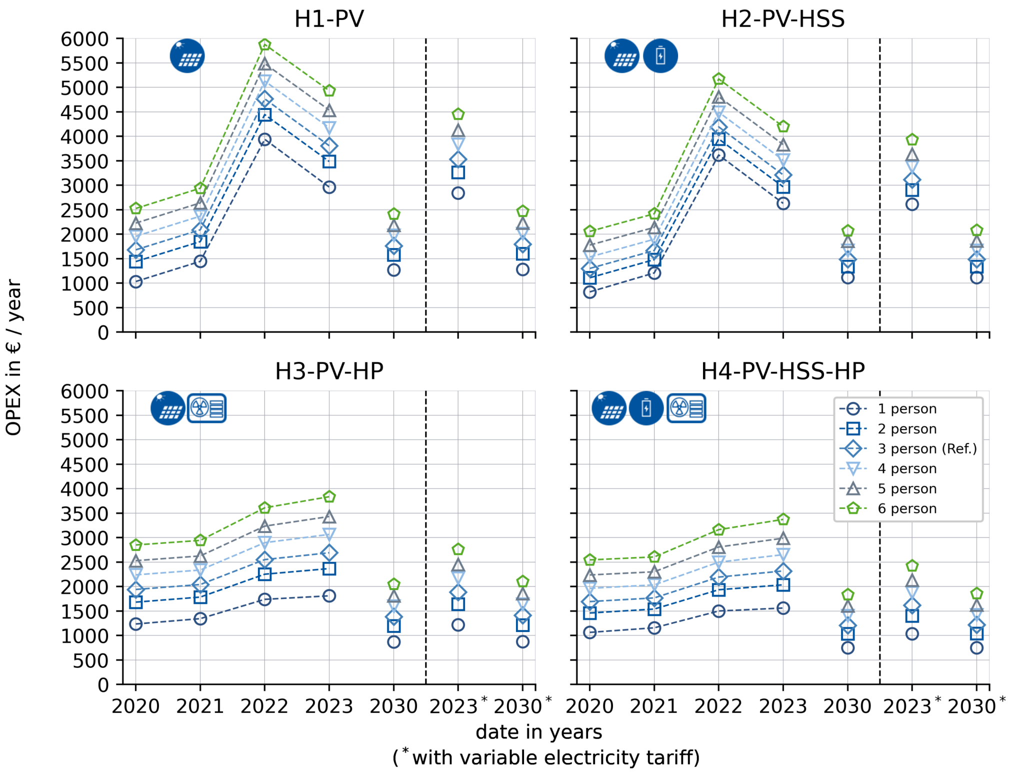

(a) Variation of number of residents, for a building constructed between 1979 and 1990.

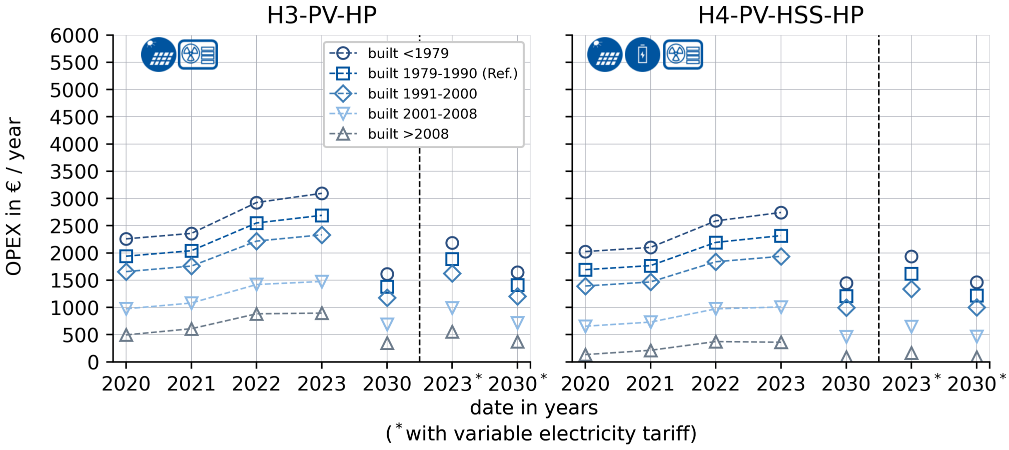

(b) Variation of years of construction of a building, for a three-person household.

Figure 6: operational expenditure (OPEX) for different renewable SFH topologies. For selected *years**, variable instead of fixed electricity tariffs are applied.

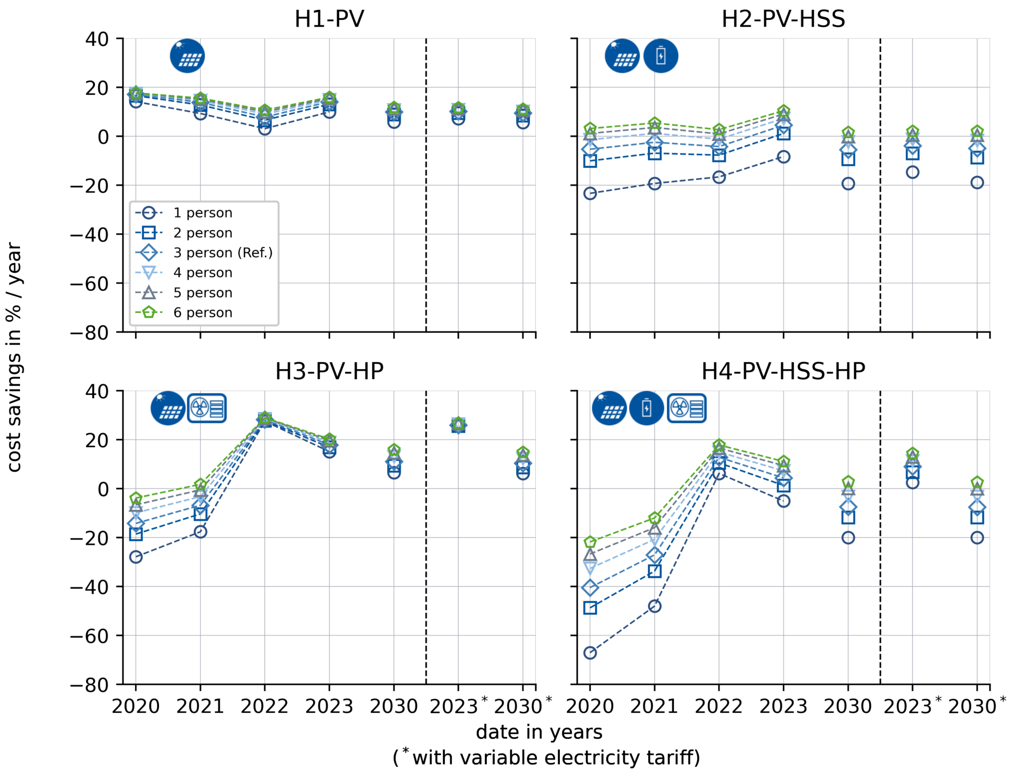

(a) Variation of number of residents, for a building constructed between 1979 and 1990.

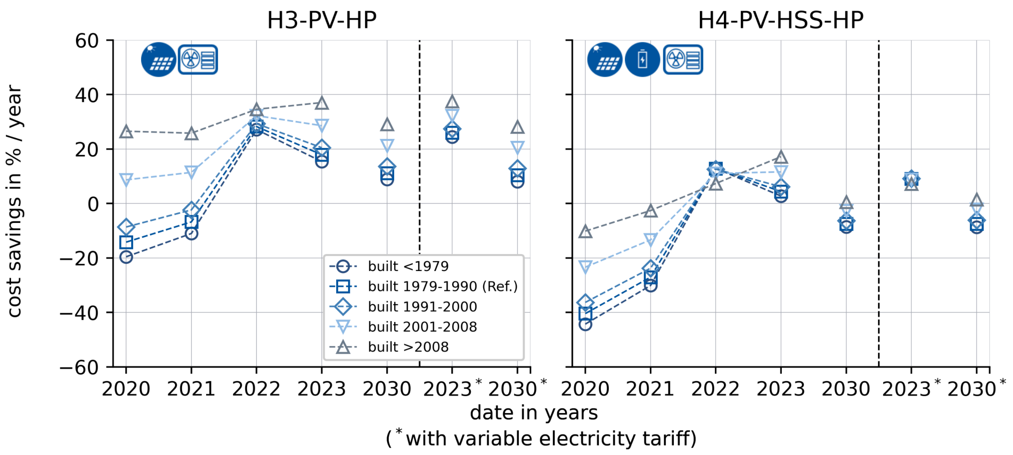

(b) Variation of years of construction of a building, for a three-person household.

Figure 7: Savings that can be achieved for different SFH topologies, compared to a standard fossil household utilizing natural gas (NG) for heating purposes. For selected $years^*$, variable instead of fixed electricity tariffs are applied.

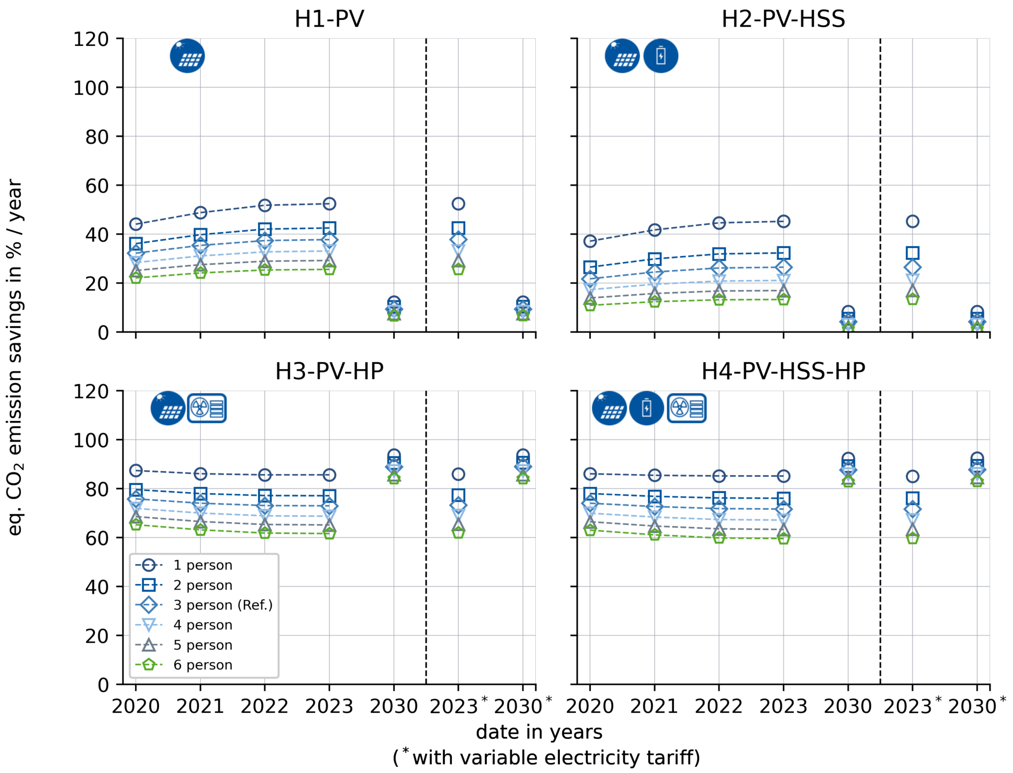

(a) Variation of number of residents, for a building constructed between 1979 and 1990.

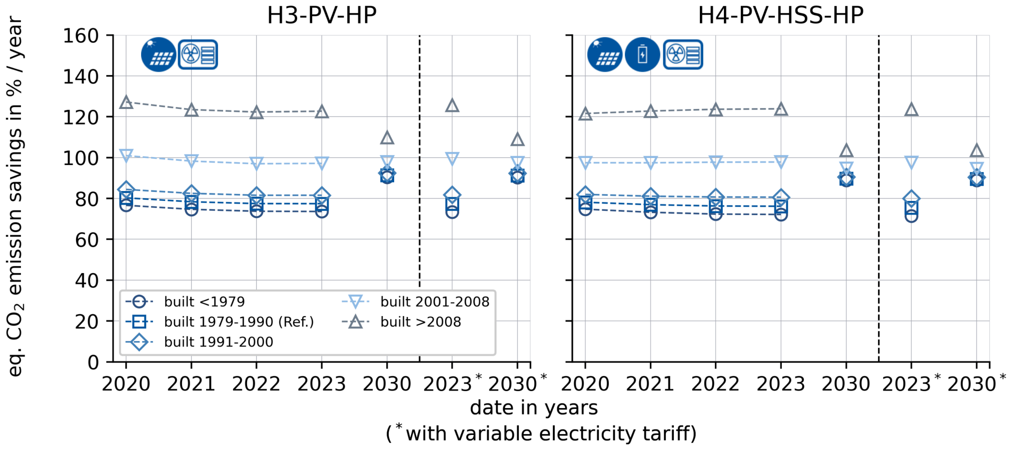

(b) Variation of years of construction of a building, for a three-person household.

Figure 8: Equivalent CO_2 reductions per kWh of consumed energy that can be achieved for different SFH topologies, compared to a standard fossil household utilizing NG for heating purposes. For selected *years**, variable instead of fixed electricity tariffs are applied.

6. Source data

Source data Figure 2a

cost savings in € / year

H1-PV	2020	2021	2022	2023	2030	2023*	2030*
1 person	362.71	263.98	163.98	443.75	142.35	301.80	137.11
2 person	524.33	440.77	397.79	688.17	259.48	454.10	249.12
3 person (Ref.)	605.61	529.69	515.38	811.10	318.39	530.18	305.01
4 Person	694.69	627.13	644.25	945.81	382.94	613.10	365.88
5 Person	775.02	715.00	760.45	1067.29	441.15	687.46	420.42
6 Person	858.10	805.88	880.64	1192.93	501.36	763.94	476.45

H3-PV-HP	2020	2021	2022	2023	2030	2023*	2030*
1 person	-714.72	-501.00	1458.32	674.40	161.53	1075.57	151.15
2 person	-587.70	-357.30	1686.68	886.75	272.64	1230.35	255.01
3 person (Ref.)	-502.52	-260.34	1848.22	1027.71	357.75	1348.56	336.17
4 Person	-393.74	-139.87	2033.46	1194.76	466.05	1489.93	440.48
5 Person	-291.99	-25.22	2209.41	1353.84	569.96	1625.97	539.70
6 Person	-187.43	90.35	2389.61	1514.68	677.23	1760.65	638.86

H2-PV-HSS	2020	2021	2022	2023	2030	2023*	2030*
1 person	-598.53	-547.54	-880.68	-370.19	-475.23	-616.22	-465.37
2 person	-318.16	-238.60	-468.84	59.54	-272.30	-332.53	-255.75
3 person (Ref.)	-187.50	-94.67	-276.93	259.95	-177.44	-201.20	-158.52
4 Person	-56.32	49.98	-84.14	460.79	-82.51	-70.50	-62.03
5 Person	50.58	167.56	72.05	624.33	-5.30	35.33	16.14
6 Person	152.85	279.83	221.63	780.66	69.16	135.29	89.63

H4-PV-HSS-HP	2020	2021	2022	2023	2030	2023*	2030*
1 person	-1719.49	-1363.57	328.28	-226.66	-493.27	107.85	-494.22
2 person	-1541.32	-1162.87	637.08	68.66	-344.31	322.00	-345.27
3 person (Ref.)	-1429.72	-1037.31	838.66	250.71	-240.21	468.52	-242.37
4 Person	-1295.96	-887.23	1063.53	459.41	-112.97	638.65	-114.87
5 Person	-1175.04	-751.54	1268.51	648.18	4.34	797.11	0.69
6 Person	-1058.13	-621.08	1469.69	830.26	120.90	949.30	114.06

Source data Figure 2b

cost savings in € / year

H3-PV-HP	2020	2021	2022	2023	2030	2023*	2030*
built <1979	-745.48	-457.91	1963.85	967.10	307.98	1405.03	282.22
built 1979-1990 (Ref.)	-502.52	-260.34	1848.22	1027.71	357.75	1348.56	336.17
built 1991-2000	-285.04	-84.77	1733.13	1075.26	400.81	1292.18	382.30
built 2001-2008	231.54	321.87	1394.66	1143.50	484.92	1111.37	472.51
built >2008	586.47	590.39	1080.13	1133.12	520.71	938.15	508.82

H4-PV-HSS-HP	2020	2021	2022	2023	2030	2023*	2030*
built <1979	-1686.24	-1249.44	935.27	169.60	-301.15	510.55	-307.62
built 1979-1990 (Ref.)	-1429.72	-1037.31	838.66	250.71	-240.21	468.52	-242.37
built 1991-2000	-1196.00	-843.88	745.72	321.40	-186.12	430.43	-182.90
built 2001-2008	-626.35	-378.84	475.71	464.01	-62.47	305.30	-50.54
built >2008	-225.09	-60.44	228.10	525.03	8.48	180.28	25.39

Source data Figure 3				
cost savings in € / year				
	H1-PV	H2-PV-HSS	H3_PV-HP	H4-PV-HSS-HP
with price breaks	811.10	259.95	1027.71	250.71
without price breaks	936.62	488.59	1712.37	993.49

Source data Figure 4a

eq. CO2 emission reductions in g / kWh / year

H1-PV	2020	2021	2022	2023	2030	2023*	2030*
1 person	107.54	120.20	123.43	123.43	31.12	123.46	31.13
2 person	88.67	99.21	101.90	101.90	25.32	101.95	25.34
3 person (Ref.)	79.48	88.98	91.41	91.41	22.52	91.47	22.54
4 Person	70.41	78.89	81.05	81.05	19.75	81.12	19.78
5 Person	62.82	70.44	72.38	72.38	17.45	72.46	17.48
6 Person	55.62	62.43	64.17	64.17	15.26	64.25	15.30

H3-PV-HP	2020	2021	2022	2023	2030	2023*	2030*
1 person	285.62	289.71	281.67	278.90	263.10	281.32	262.80
2 person	259.71	263.21	255.53	252.93	244.70	254.96	244.50
3 person (Ref.)	247.38	250.56	242.89	240.39	236.21	242.30	236.06
4 Person	234.83	237.69	230.09	227.63	227.41	229.49	227.29
5 Person	224.20	226.78	219.35	216.92	219.91	218.64	219.81
6 Person	213.82	216.13	208.86	206.49	212.43	208.08	212.35

H2-PV-HSS	2020	2021	2022	2023	2030	2023*	2030*
1 person	83.44	93.91	96.56	96.56	20.70	96.63	20.71
2 person	57.85	65.47	67.41	67.40	12.96	67.48	12.98
3 person (Ref.)	47.12	53.52	55.15	55.12	9.77	55.21	9.80
4 Person	37.49	42.75	44.08	44.08	6.95	44.19	6.97
5 Person	30.30	34.73	35.85	35.82	4.89	35.95	4.90
6 Person	23.99	27.70	28.61	28.56	3.07	28.74	3.11

H4-PV-HSS-HP	2020	2021	2022	2023	2030	2023*	2030*
1 person	268.46	274.53	267.50	264.61	255.13	265.04	255.10
2 person	240.43	244.89	237.92	235.21	236.19	235.63	236.16
3 person (Ref.)	227.39	231.16	224.15	221.50	227.61	221.94	227.60
4 Person	214.30	217.39	210.42	207.85	218.75	208.24	218.74
5 Person	203.69	206.22	199.27	196.77	211.32	197.08	211.34
6 Person	193.67	195.68	188.86	186.42	204.07	186.71	204.06

Source data Figure 4b

eq. CO2 emission reductions in g / kWh / year

H3-PV-HP	2020	2021	2022	2023	2030	2023*	2030*
built <1979	209.60	209.54	202.25	199.91	228.78	199.12	228.57
built 1979-1990 (Ref.)	222.63	223.62	216.50	214.35	228.81	214.14	228.54
built 1991-2000	237.82	239.91	232.81	230.72	228.85	231.57	228.48
built 2001-2008	304.17	309.71	302.51	300.90	228.93	307.62	228.10
built >2008	430.27	445.98	442.86	442.36	229.27	453.30	227.63

H4-PV-HSS-HP	2020	2021	2022	2023	2030	2023*	2030*
built <1979	204.46	205.48	198.43	195.96	225.07	194.16	224.80
built 1979-1990 (Ref.)	216.70	219.55	213.22	210.84	224.60	209.01	224.31
built 1991-2000	230.95	235.89	230.38	228.08	224.04	226.24	223.74
built 2001-2008	293.66	307.04	304.91	302.97	221.47	302.04	221.15
built >2008	411.34	443.48	447.75	446.54	216.34	446.11	216.21

Source data Figure 5a

cost savings in € / year

	2020	2021	2022	2023	2030
H1 (8.7 kWh)	605.61	529.69	515.38	811.10	318.39
H1 (13.7 kWh)	683.42	541.89	428.72	747.57	280.55
H2 (8.7 kWh)	-187.50	-94.67	-276.93	259.95	-177.44
H2 (13.7 kWh)	-68.66	-37.46	-303.43	283.87	-180.77
H3 (8.7 kWh)	-502.52	-260.34	1848.22	1027.71	357.75
H3 (13.7 kWh)	-295.28	-102.21	1964.04	1214.72	420.90
H4 (8.7 kWh)	-1429.72	-1037.31	838.66	250.71	-240.21
H4 (13.7 kWh)	-1134.13	-783.55	1079.74	583.92	-108.33

Source data Figure 5b

eq. CO2 emission reductions in g / kWh / year

	2020	2021	2022	2023	2030
H1 (8.7 kWh)	132.23	147.39	151.26	151.26	39.50
H1 (13.7 kWh)	208.22	232.10	238.19	238.19	62.20
H2 (8.7 kWh)	127.91	142.61	146.36	146.36	38.11
H2 (13.7 kWh)	203.45	226.82	232.78	232.78	60.66
H3 (8.7 kWh)	224.39	225.39	218.36	216.25	230.74
H3 (13.7 kWh)	298.80	304.11	299.41	297.53	252.88
H4 (8.7 kWh)	221.90	224.77	218.52	216.18	229.96
H4 (13.7 kWh)	296.02	305.25	301.25	299.17	252.07

Source data Figure 5c

cost savings in € / year

	2020	2021	2022	2023	2030
H1	605.61	529.69	515.38	811.10	318.39
H1 + EV	690.92	623.01	638.79	940.11	380.21
H2	-187.50	-94.67	-276.93	259.95	-177.44
H2 + EV	63.75	183.46	95.87	648.33	4.55
H3	-502.52	-260.34	1848.22	1027.71	357.75
H3 + EV	-436.49	-188.27	1944.40	1128.16	405.62
H4	-1429.72	-1037.31	838.66	250.71	-240.21
H4 + EV	-1245.55	-833.28	1112.08	536.49	-105.33

Source data Figure 5d					
cost savings in € / year		H1	H2	H3	H4
	IR 2% (default)	811.10	259.95	1027.71	250.71
	IR 3%	732.08	107.23	892.83	43.35
	IR 4%	651.30	-48.14	751.84	-170.90
	IR 5%	568.81	-206.22	607.70	-388.96

References

[1] J. Figgener, "Speichermonitoring bw: Jahresbericht 2022." [Online]. Available: https://www.researchgate.net/publication/362861071_Speichermonitoring_BW_20_Schlussbericht_inhaltlicher_Teil/link/63048aacaa4b1206facf1d3c/download

[2] H. Wirth, "Recent facts about photovoltaics in germany." [Online]. Available: https://www.ise.fraunhofer.de/en/publications/studies/recent-facts-about-pv-in-germany.html

[3] International Renewable Energy Agency, "Electricity storage and renewables: Costs and markets to 2030." [Online]. Available: https://www.irena.org/-/media/Files/IRENA/Agency/Publication/2017/Oct/IRENA_Electricity_Storage_Costs_2017.pdf

[4] J. Figgener, D. Haberschusz, K.-P. Kairies, O. Wessels, B. Tepe, and D. U. Sauer, "Wissenschaftliches mess- und evaluierungsprogramm solarstromspeicher 2.0: Jahresbericht 2018." [Online]. Available: https://www.researchgate.net/publication/326507444_Wissenschaftliches_Mess-und_Evaluierungsprogramm_Solarstromspeicher_20_Jahresbericht_2018?channel=doi&linkId=5b5192a245851507a7b218bb&showFulltext=true

[5] J. Figgener, C. Hecht, J. Bors, K. Spreuer, K.-P. Kairies, P. Stenzel, and D. U. Sauer, "The development of battery storage systems in germany: A market review (status 2023)," 2023. [Online]. Available: https://www.researchgate.net/publication/369479477_The_development_of_battery_storage_systems_in_Germany_A_market_review_status_2023

[6] J. M. Mayer, S. Philipps, N. S. Hussein, T. Schlegl, and C. Senkpiel, "Current and future cost of photovoltaics: Long-term scenarios for market development, system prices and lcoe of utility-scale pv systems." [Online]. Available: https://www.ise.fraunhofer.de/content/dam/ise/de/documents/publications/studies/AgoraEnergiewende_Current_and_Future_Cost_of_PV_Feb2015_web.pdf

[7] M. Taylor, P. Ralon, T. Nowak, and C. Dittmar, "Renewable solutions in end-uses: Heat pump costs and markets," Abu Dhabi. [Online]. Available: https://www.irena.org/-/media/Files/IRENA/Agency/Publication/2022/Nov/IRENA_Heat_Pumps_Costs_Markets_2022.pdf

[8] D. Lencz and J. Wagner, "Energiemarkt 2030 und 2050 - der beitrag von gas- und wärmeinfrastruktur zu einer effizienten co2-minderung." [Online]. Available: https://www.ewi.uni-koeln.de/cms/wp-content/uploads/2017/11/ewi_ERS_Energiemarkt_2030_2050.pdf

[9] Fraunhofer Institute for Solar Energy Systems, "Auch in bestandsgebäuden funktionieren wärmepumpen zuverlässig und sind klimafreundlich – feldtest des fraunhofer ise abgeschlossen," 07/2020. [Online]. Available: https://www.ise.fraunhofer.de/content/dam/ise/de/documents/presseinformationen/2020/1920_ISE_d_PI_Abschluss_WPsmartimBestand.pdf

[10] Techem Energy Services GmbH, "Techem verbrauchskennwerte 2022: Erhebungen und analysen zum energieverbrauch und zur co2-emission für heizung und warmwasser in deutschen mehrfamilienhäusern." [Online]. Available: https://www.techem.com/content/dam/techem/downloads/techem-com/vkw-studie/23-44-001%20VKW%202022%20Leseversion.pdf.coredownload.inline.pdf

[11] M. Lödl, G. Kerber, R. Witzmann, C. Hoffmann, and M. Metzger, "Abschätzung des photovoltaik-potentials auf dachflächen in deutschland," Graz, 10.02.2010. [Online]. Available: https://mediatum.ub.tum.de/doc/%20969497/969497.pdf

[12] Bundesinstitut für Bau-, Stadt- und Raumforschung, "Nutzenergiebedarf für warmwasser in wohngebäuden." [Online]. Available: https://www.bbsr.bund.de/BBSR/DE/veroeffentlichungen/bbsr-online/2017/bbsr-online-17-2017-dl.pdf?__blob=publicationFile&v=1

[13] European Association for Storage of Energy, "Thermal hot water storage." [Online]. Available: https://ease-storage.eu/wp-content/uploads/2016/03/EASE_TD_HotWater.pdf

[14] H. Bechem, M. Blesl, M. Brunner, J. Conrad, T. Falke, C. Felsmann, M. Geipel, N. Gerhardt, W. Glaunsinger, J. Hilpert, A. Kessler, M. Kleimaier, S. Köhler, M. Lüking, P. Mayrhofer, A. Meinzenbach, E. Metten, H. Neugebauer, D. Oesterwind, and B. Wille-Haussmann, *Potenziale für Strom im Wärmemarkt bis 2050: Wärmeversorgung in flexiblen Energieversorgungssystemen mit hohen Anteilen an erneuerbaren Energien.* Association for Electrical, Electronic & Information Technologies (VDE), 2015. [Online]. Available: https://docplayer.org/24584807-Potenziale-fuer-strom-im-waermemarkt-bis-2050.html

[15] A. A. Kebede, T. Kalogiannis, J. van Mierlo, and M. Berecibar, “A comprehensive review of stationary energy storage devices for large scale renewable energy sources grid integration,” *Renewable and Sustainable Energy Reviews*, vol. 159, p. 112213, 2022. [Online]. Available: https://www.sciencedirect.com/science/article/pii/S1364032122001368

[16] D. Günther, J. Wapler, R. Langner, S. Helmling, M. Miara, D. Fischer, D. Zimmermann, T. Wolf, and B. Wille-Hausmann, “Wärmepumpen in bestandsgebäuden: Abschlussbericht - ergebnisse aus dem projekt wpsmart im bestand.” [Online]. Available: https://www.ise.fraunhofer.de/content/dam/ise/de/downloads/pdf/Forschungsprojekte/BMWi-03ET1272A-WPsmart_im_Bestand-Schlussbericht.pdf

[17] A. Müller, L. Friedrich, C. Reichel, S. Herceg, M. Mittag, and D. H. Neuhaus, “A comparative life cycle assessment of silicon pv modules: Impact of module design, manufacturing location and inventory,” *Solar Energy Materials and Solar Cells*, vol. 230, p. 111277, 2021. [Online]. Available: https://www.sciencedirect.com/science/article/pii/S0927024821003202

[18] E. Emilsson and L. Dahllöf, “Lithium-ion vehicle battery production status 2019 on energy use, co 2 emissions, use of metals, products environmental footprint, and recycling.” [Online]. Available: https://www.ivl.se/download/18.34244ba71728fcb3f3faf9/1591706083170/C444.pdf

[19] G. Naumann, E. Schropp, and M. Gaderer, “Life cycle assessment of an air-source heat pump and a condensing gas boiler using an attributional and a consequential approach,” *Procedia CIRP*, vol. 105, pp. 351–356, 2022. [Online]. Available: https://www.sciencedirect.com/science/article/pii/S2212827122000580

[20] L. Krebs, R. Frischknecht, P. Stolz, and P. Sinha, “Environmental life cycle assessment of residential pv and battery storage systems 2020.” [Online]. Available: https://iea-pvps.org/wp-content/uploads/2020/07/IEA_PVPS_Task12_LCA_PVandStorage.pdf

[21] J. Gong, Y. Nie, J. van Ouwerkerk, F. Wege, M. C. Cortés, C. v. Oy, J. Brucksch, C. Bußar, T. Schreiber, D. U. Sauer, D. Müller, and A. Monti, “Focus : A framework for energy system optimization from prosumer to district and city scale.” [Online]. Available: https://arxiv.org/abs/2304.07150

[22] T. Tjaden, B. Bergner, J. Weniger, and V. Quaschning, “Repräsentative elektrische lastprofile für einfamilienhäuser in deutschland auf 1-sekündiger datenbasis: Datensatz,” Berlin. [Online]. Available: https://solar.htw-berlin.de/wp-content/uploads/HTW-Repraesentative-elektrische-Lastprofile-fuer-Wohngebaeude.pdf

[23] D. Fischer, T. Wolf, J. Scherer, and B. Wille-Haussmann, “A stochastic bottom-up model for space heating and domestic hot water load profiles for german households,” *Energy and Buildings*, vol. 124, pp. 120–128, 2016. [Online]. Available: https://www.sciencedirect.com/science/article/pii/S0378778816303358

[24] Fraunhofer Institute for Solar Energy Systems ISE, “Energy-charts,” 2023. [Online]. Available: https://energy-charts.info/index.html?l=de&c=DE

[25] S. Pfenninger and I. Staffell, “Renewables.ninja.” [Online]. Available: https://www.renewables.ninja/

[26] R. Gelaro, W. McCarty, M. J. Suárez, R. Todling, A. Molod, L. Takacs, C. A. Randles, A. Darmenov, M. G. Bosilovich, R. Reichle, K. Wargan, L. Coy, R. Cullather, C. Draper, S. Akella, V. Buchard, A. Conaty, A. M. d. Silva, W. Gu, G.-K. Kim, R. Koster, R. Lucchesi, D. Merkova, J. E. Nielsen, G. Partyka, S. Pawson, W. Putman, M. Rienecker, S. D. Schubert, M. Sienkiewicz, and B. Zhao, “The modern-era retrospective analysis for research and applications, version 2 (merra-2),” *Journal of Climate*, vol. 30, no. 14, pp. 5419–5454, 2017. [Online]. Available: https://journals.ametsoc.org/view/journals/clim/30/14/jcli-d-16-0758.1.xml

[27] S. Pfenninger and I. Staffell, “Long-term patterns of european pv output using 30 years of validated hourly reanalysis and satellite data,” *Energy*, vol. 114, pp. 1251–1265, 2016. [Online]. Available: https://www.sciencedirect.com/science/article/pii/S0360544216311744

[28] Deutscher Wetterdienst, “Ortsgenaue testreferenzjahre von deutschland für mittlere, extreme und zukünftige witterungsverhältnisse: Handbuch,” Offenbach. [Online]. Available: https://www.bbsr.bund.de/BBSR/DE/forschung/programme/zb/Auftragsforschung/5EnergieKlimaBauen/2013/testreferenzjahre/try-handbuch.pdf;jsessionid=32F96E889EBF9043C884BC440CF32493.live21301?__blob=publicationFile&v=1

[29] F. Rücker, M. Merten, J. Gong, R. Villafáfila-Robles, I. Schoeneberger, and D. U. Sauer, “Evaluation of the effects of smart charging strategies and frequency restoration reserves market participation of an electric vehicle,” *Energies*, vol. 13, no. 12, 2020. [Online]. Available: https://www.mdpi.com/1996-1073/13/12/3112

[30] F. Hein and H. Hauke, "Agorameter: Dokumentation." [Online]. Available: https://static.agora-energiewende.de/fileadmin/Projekte/Agorameter/A-EW_Hintergrunddokumentation_Agorameter_v37_web.pdf

[31] A. Bett and B. Burger, "Photovoltaics report." [Online]. Available: https://www.ise.fraunhofer.de/content/dam/ise/de/documents/publications/studies/Photovoltaics-Report.pdf

[32] K. Hansen, "Decision-making based on energy costs: Comparing levelized cost of energy and energy system costs," *Energy Strategy Reviews*, vol. 24, pp. 68–82, 2019. [Online]. Available: https://www.sciencedirect.com/science/article/pii/S2211467X19300197

[33] D. Ritter and D. Bauknecht, "Wirtschaftlichkeit von photovoltaik-dachanlagen: Eine differenzierte betrachtung von volleinspeise- und eigenverbrauchsanlagen." [Online]. Available: https://www.umweltbundesamt.de/sites/default/files/medien/479/publikationen/cc_66-2021_wirtschaftlichkeit_von_photovoltaik-dachanlagen.pdf

[34] H. Krützfeldt, C. Vering, P. Mehrfeld, and D. Mueller, "Milp design optimization of heat pump systems in german residential buildings," *Energy and Buildings*, vol. 249, p. 111204, 2021.

[35] H.-M. Henning and A. Palzer, "Was kostet die energiewende? wege zur transformation des deutschen energiesystems bis 2050," Freiburg. [Online]. Available: https://www.fraunhofer.de/content/dam/zv/de/Forschungsfelder/Energie-Rohstoffe/Fraunhofer-ISE_Transformation-Energiesystem-Deutschland_final_19_11%20(1).pdf

[36] R. Gross and R. Hanna, "Path dependency in provision of domestic heating," *Nature Energy*, vol. 4, no. 5, pp. 358–364, 2019. [Online]. Available: https://doi.org/10.1038/s41560-019-0383-5

[37] K. B. Lindberg, D. Fischer, G. Doorman, M. Korpås, and I. Sartori, "Cost-optimal energy system design in zero energy buildings with resulting grid impact: A case study of a german multi-family house," *Energy and Buildings*, vol. 127, pp. 830–845, 2016. [Online]. Available: https://www.sciencedirect.com/science/article/pii/S0378778816304327

[38] R. Galvin, "Why german households won't cover their roofs in photovoltaic panels: And whether policy interventions, rebound effects and heat pumps might change their minds," *Renewable Energy Focus*, vol. 42, pp. 236–252, 2022. [Online]. Available: https://www.sciencedirect.com/science/article/pii/S1755008422000540

[39] L. Tschümperlin, P. Stolz, and R. Frischknecht, "Life cycle assessment of low power solar inverters (2.5 to 20 kw)," *Swiss Federal Office of Energy SFOE*, 2016. [Online]. Available: https://treeze.ch/fileadmin/user_upload/downloads/Publications/Case_Studies/Energy/174-Update_Inverter_IEA_PVPS_v1.1.pdf

[40] K. Mongird, V. Viswanathan, P. Balducci, J. Alam, V. Fotedar, V. Koritarov, and B. Hadjerioua, "An evaluation of energy storage cost and performance characteristics," *Energies*, vol. 13, no. 13, 2020. [Online]. Available: https://www.mdpi.com/1996-1073/13/13/3307

[41] Sonnen, "Technische daten sonnenbatterie 10." [Online]. Available: https://cdn-sonnen-media.s3.amazonaws.com/8c6fa3c1-e788-41e4-818d-c8e4b2d4833b-de

[42] Nico Orth, Nina Munzke, Johannes Weniger, Christian Messner, Robert Schreier, Michael Mast, Lucas Meissner, and Volker Quaschning, "Efficiency characterization of 26 residential photovoltaic battery storage systems," *Journal of Energy Storage*, vol. 65, p. 107299, 2023. [Online]. Available: https://www.sciencedirect.com/science/article/pii/S2352152X23006965

[43] SMA Solar Technology AG, "Technische information wirkungsgrade und derating sunny boy / sunny tripower / sunny mini central." [Online]. Available: https://www.photovoltaik4all.de/media/pdf/34/d0/a1/WirkungDerat-TI-de-40.pdf

[44] J. Knoefel and B. Herrmann, "Technisch-ökonomische bewertung von quartierspeichern," 2021. [Online]. Available: https://www.ioew.de/fileadmin/user_upload/BILDER_und_Downloaddateien/Publikationen/2021/Knoefel_Herrmann_2021_Technisch_oekonomische_Bewertung_von_Quartierspeichern.pdf
